# Anion Exchange Membrane with Pendulous Piperidinium on Twisted All-Carbon Backbone for Fuel Cell

**DOI:** 10.3390/membranes14060121

**Published:** 2024-05-23

**Authors:** Huaqing Zhang, Wanjie Song, Lixuan Sun, Cui Yang, Xin Zhang, Mingyue Wu, Liang Wu, Xiaolin Ge, Tongwen Xu

**Affiliations:** Key Laboratory of Precision and Intelligent Chemistry, School of Chemistry and Materials Science, University of Science and Technology of China, Hefei 230026, China; zhanghq1@mail.ustc.edu.cn (H.Z.); swanjie@mail.ustc.edu.cn (W.S.); sunlixuan@mail.ustc.edu.cn (L.S.); yangcui1020@mail.ustc.edu.cn (C.Y.); aa123456@mail.ustc.edu.cn (X.Z.); mingyue_wu@mail.ustc.edu.cn (M.W.); liangwu8@ustc.edu.cn (L.W.)

**Keywords:** anion exchange membrane, all-carbon backbone, twisted structure, free volume, fuel cell

## Abstract

As a central component for anion exchange membrane fuel cells (AEMFCs), the anion exchange membrane is now facing the challenge of further improving its conductivity and alkali stability. Herein, a twisted all-carbon backbone is designed by introducing stereo-contorted units with piperidinium groups dangled at the twisted sites. The rigid and twisted backbone improves the conduction of hydroxide and brings down the squeezing effect of the backbone on piperidine rings. Accordingly, an anion exchange membrane prepared through this method exhibits adapted OH^−^ conductivity, low swelling ratio and excellent alkali stability, even in high alkali concentrations. Further, a fuel cell assembled with a such-prepared membrane can reach a power density of 904.2 mW/cm^2^ and be capable of continuous operation for over 50 h. These results demonstrate that the designed membrane has good potential for applications in AEMFCs.

## 1. Introduction

The development and utilization of hydrogen energy represents a significant step towards achieving carbon reduction and energy sustainability [1,2]. The fuel cell is a convenient, safe and efficient technology for utilizing hydrogen energy. In particular, anion exchange membrane fuel cells (AEMFCs) are attracting increasing attention due to their economic efficiency and compatibility with inexpensive metal catalysts [2,3]. As a central component, the anion exchange membrane (AEM) exerts a direct influence on the performance and lifetime of AEMFCs [3,4,5]. An ideal AEM should provide good conductivity, low swelling ratio and high mechanical and chemical stability [2,3,4]. However, a majority of the current AEMs are facing a trade-off between swelling ratio, conductivity and chemical stability [4,6].

In general, sufficient ion exchange capacity (IEC) is essential to guarantee the ion transport ability of the membrane. However, an increase in the IEC is accompanied by excessive water uptake and thus more swelling in the membrane, which impairs suitability for application and stability [7,8]. Therefore, it is imperative to enhance conductivity while maintaining a low swelling ratio. Over the past decade, a number of polymer structures have been developed with the objective of facilitating microphase separation in the membrane [8,9,10,11,12,13,14,15,16,17], such as graft structure [14], comb structure [12,13] and block structure [15,17]. These structures can facilitate microphase separation and construction of ion channels in the membrane, thereby achieving high conductivity at low IEC. In addition, recent studies have indicated that the introduction of twisted units or the construction of branched structures can increase the free volume in polymers, thereby enhancing ion transport and reducing swell ratio of the membrane [18,19,20,21,22,23,24,25].

Deterioration in the AEM performance in an alkaline environment is primarily attributed to the fracture of polymer backbone and degradation of ion exchange groups [26,27,28]. With regard to the former, numerous studies have demonstrated that the aryl ether bonds in conventional poly(aryl ether) polymers are susceptible to OH^−^ attack, resulting in severe degradation of the polymers [29,30,31,32,33,34,35,36]. To address this issue, a series of all-carbon backbone polymers have been synthesized based on the superacid catalysis reaction, which encourage high chemical stability and emerge as promising candidates for the next generation of AEMs [24,37,38,39,40,41]. For ion exchange groups, a variety of cation groups have been developed and evaluated [42,43,44,45,46,47,48,49]. Among these, cyclic amines, such as piperidinium, are considered to be more stable [47,48]. In addition, the bonging methods and positions of cation groups can also influence chemical stability of the membrane. The general grafting method frequently results in the formation of sensitive sites, such as benzyl and carbonyl [35,50,51]. The Suzuki coupling reaction is a simple and effective process that enables direct construction of C-C bonds, making it an ideal candidate for the synthesis of highly stable AEMs [52,53]. Moreover, some studies have demonstrated that when piperidinium is embedded into a highly rigid backbone, the backbone exerts pressure on it, which will leave the group more susceptible to ring-opening degradation [41,53,54].

**Scheme 1:**
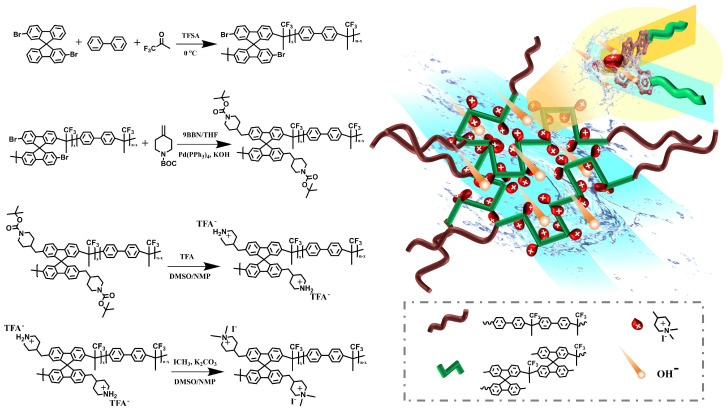
Diagram of synthesis strategy for the AEM with pendulous piperidinium on twisted all-carbon backbone.

This paper presents a novel AEM with pendulous piperidinium on a twisted all-carbon backbone. As illustrated in Scheme 1, spirobifluorene units are incorporated into a polymer backbone via the superacid catalysis reaction. The stereo-contorted units force the backbone to form a twisted structure, while piperidiniums are dangled at the twisted sites through the Suzuki coupling reaction. The twisted backbone facilitates water uptake and ion conduction. Furthermore, the piperidinium is situated outside the backbone, rather than being embedded into it. This structure can reduce the squeezing effect of the backbone on piperidinium rings, thereby improving chemical stability. Consequently, AEMs prepared based on this structure can meet the demand for conductivity (approximately 100 mS cm^−1^), exhibit a low swelling ratio (less than 10%) and achieve high chemical stability, even in a highly concentrated alkali solution. Further, a fuel cell assembled using a such-prepared membrane can reach a good power density of 904.2 mW/cm^2^ and be capable of continuous operation for over 50 h.

## 2. Result and Discussion

### 2.1. Synthesis and Structural Analysis

The all-carbon twisted backbone polymer is synthesized based on the superacid catalysis reaction. As shown in Figure S1a, signal peaks at 1.98 ppm and at 6–8 ppm are assigned to alkyl and aromatic hydrogens, respectively. The signal peak at 6.77 ppm corresponds to hydrogen on the benzene ring adjacent to bromine, proving successful introduction of the stereo-contorted unit. Piperidine groups are introduced via the Suzuki coupling reaction to avoid the generation of any sensitive polar bonds. As shown in Figure S1b, signal peaks between 6 and 8 ppm correspond to aromatic hydrogens. The successful functionalization is evidenced by the appearance of new peaks at 3.96 ppm, 2.51 ppm, 2.35 ppm, 1.59 ppm, and 1.26 ppm, which belong to the piperidine and Boc groups. The polymer is subsequently deprotected in a solvent mixture of DMSO/NMP/TFA and finally quaternized by methyl iodide. As shown in Figure S1c, the signal peak corresponding to the Boc group disappears after the deprotection reaction. The newly emerged peak at 8.0–8.5 ppm is attributed to protonated secondary amine. After quaternization, the signal peak corresponding to the protonated secondary amine disappears and a new peak appears at 3.01 ppm, which is attributed to methyl hydrogen on the quaternary amino group (Figure S1d). All these results demonstrate that the polymer is successfully synthesized with the designed structure. 

**Figure 1:**
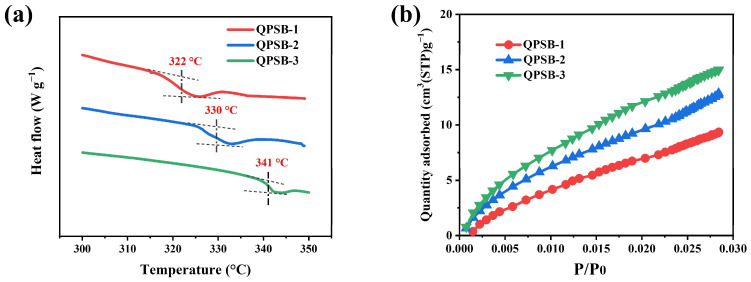
(**a**) The glass transition temperature (Tg) of QPSB membranes. (**b**) The CO_2_ adsorption (at 273 K) of QPSB membranes.

As shown in Figure 1a, the glass transition temperature (Tg) of material gradually increases from 325 °C to 343 °C as the content of stereo-contorted units increases in the polymer backbone. This is due to the fact that the stereo-contorted units force the polymer backbone to form a twisted structure, which enhances stiffness, strengthens the polymer bulk and hinders the motility of chains. As shown in Figure 1b, the CO_2_ adsorption of material increases with the inclusion of more stereo-contorted units. One potential explanation for this is that the rigid and twisted backbone may result in ineffective chain packing, which can create some free pore space. 

### 2.2. Morphology Characterization

The surface and cross-sectional morphologies of these prepared membranes are observed by scanning electron microscopy (SEM). As illustrated in Figure S2a–f, these membranes exhibit a smooth and flat surface with a uniform structure. Displaying no discernible defects, these membranes prove good processability of materials. 

**Figure 2:**
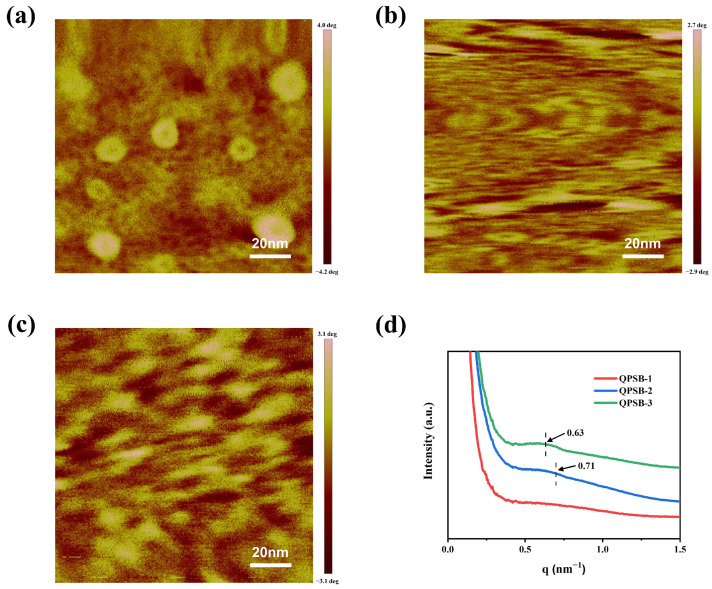
(**a**–**c**) The AFM phase images of QPSB-1, QPSB-2 and QPSB-3 membranes. (**d**) The SAXS profiles of different membranes.

As shown in Figure 2a–c, the microphase separation morphologies of prepared AEMs are characterized by atomic force microscopy (AFM). The darker areas indicate the hydrophilic regions, and the lighter areas indicate the hydrophobic regions in the membrane. It is evident that the QPSB-1 membrane exhibits discrete and smaller ionic clusters due to its low IEC. As the contents of stereo-contorted units and ion exchange groups increase, the ionic clusters increase significantly in scale, which accordingly form highly connected hydrophilic channels.

In order to further study the microphase separation properties of these membranes, small angle X-ray scattering (SAXS) analysis is conducted. As shown in Figure 2d, the QPSB-1 membrane does not induce significant signals, whereas QPSB-2 corresponds to an obvious signal peak with a q value of 0.71 nm^−1^. The QPSB-3 membrane shows a more pronounced signal peak with a smaller q value of 0.63 nm^−1^. The q value in SAXS is indicative of the size of the ionic cluster. The smaller q value indicates larger ionic clusters and the presence of larger channels. The more pronounced signal peak demonstrates the formation of a more long-range ordered microphase separation morphology. These results are in alignment with the AFM characteristics.

### 2.3. Ion Exchange Capacity (IEC), Water Uptake (WU) and Swelling Ratio (SR)

**Figure 3:**
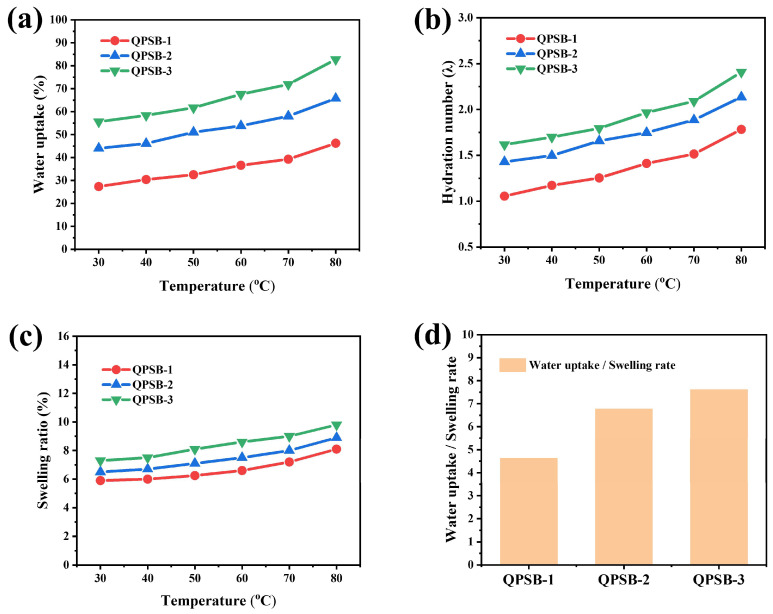
(**a**) The water uptakes of different membranes at varied temperatures. (**b**) The hydration number of membranes at different temperatures. (**c**) The swelling ratio of different membranes at varied temperatures. (**d**) The ratio of water uptake to swelling of QPSB-1, QPSB-2 and QPSB-3 membranes.

The ion exchange capacity of the membrane is determined by the content of ion exchange groups, which directly affects the membrane’s water uptake, swelling ratio and conductivity. As shown in Table S1, the IEC of QPSB membranes ranges from 1.38 mmol g^−1^ to 1.81 mmol g^−1^, depending on the quantity of stereo-contorted units. The water absorption properties of the membrane influences conductivity and dimensional stability. As shown in Figure 3a, the water uptake of these membranes continually increase as the content of stereo-contorted units grows. This is mainly attributed to the rise in IEC. In addition, the twisted backbone structure prevents tight packing of chains, which can also promote water uptake.

Moreover, piperidine groups in the membrane are deliberately dangled at the twisted sites, away from the hydrophobic backbone, which therefore can be more readily solvated. This hypothesis can be corroborated by examining the hydration number (λ) of groups in different membranes. As shown in Figure 3b, the λ is defined as the number of water molecules per mol of charged functional groups. It is evident that the membrane with a high proportion of stereo-contorted units has a higher λ.

The swelling ratio can lead to dimensional changes in the membrane, which impedes its application. So, it is imperative to restrict the extent of membrane swelling to a reasonable range. As shown in Figure 3c, the swelling ratios of all membranes remain below 8% at room temperature and do not exceed 10% even at 80 °C, demonstrating excellent dimensional stability. Furthermore, it can be observed that as the content of stereo-contorted units increased, the ratio of water uptake to swelling gradually increases (Figure 3d). This is to say that a membrane with a high content of stereo-contorted units enjoys a higher capacity to absorb water at a given swelling ratio. This can be attributed to the looser packing of the highly twisted polymer chains.

### 2.4. Hydroxide Conductivity

**Figure 4:**
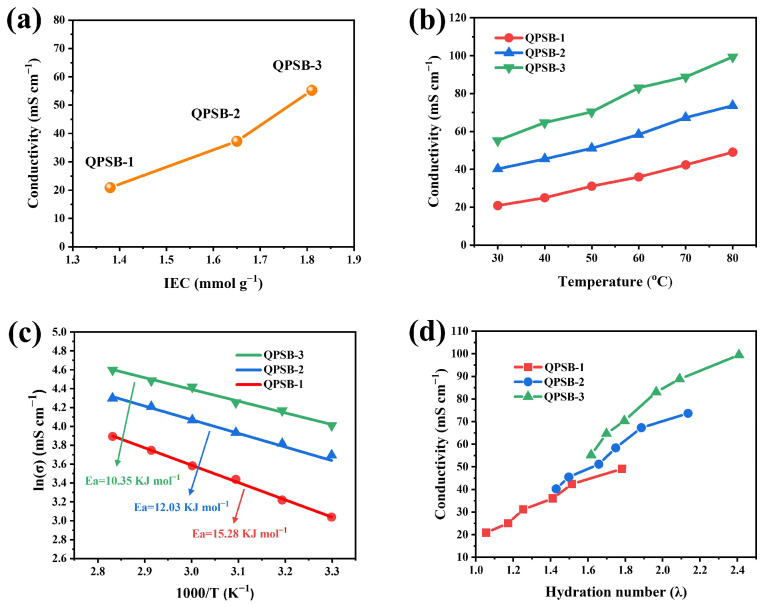
(**a**) The OH^−^ conductivity of membranes with different IEC at 30 °C. (**b**) The OH^−^ conductivity as a function of temperature of different membranes. (**c**) The Arrhenius plots and activation energy of different membranes. (**d**) The OH^−^ conductivity as a function of the hydration number of different membranes.

Hydroxide conductivity represents a crucial indicator for evaluating AEMs, as it directly affects the performance of AEMFCs. As shown in Figure 4a, an increase in the content of stereo-contorted units is accompanied by a notable enhancement in membrane conductivity, due to elevated IEC and enhanced water absorption. Figure 4b presents conductivity variations with temperature for different membranes. Notably, the QPSB-3 membrane exhibits the highest conductivity which increases from 55.2 mS cm^−1^ to 99.4 mS cm^−1^ when the temperature is from 30 °C to 80 °C. 

The change in conductivity with temperature can be accounted for by the ion transport activation energy (Ea). As shown in Figure 4c, the calculated Ea values of QPSB-1, QPSB-2 and QPSB-3 are 15.28 kJ mol^−1^, 12.03 kJ mol^−1^ and 10.35 kJ mol^−1^, respectively. It is evident that the introduction of stereo-contorted units results in a reduction in the activation energy required for ion transport. This is the consequence of the combined impact of elevated IEC, enhanced microphase separation and improved water absorption. Figure 4d shows the correlation between the hydration number and conductivity. Apparently, the QPSB-3 membrane manifests a higher ratio between conductivity and hydration number than QPSB-1 and QPSB-2. This indicates that the membrane is more efficient as a hydroxide ion conductor and confirms the advantage of positioning piperidine cations at the twisted sites instead of into the stiff backbone.

### 2.5. Mechanical, Thermal and Chemical Stability

**Figure 5:**
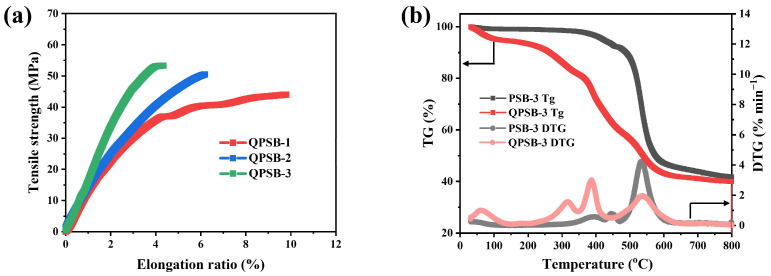
(**a**) The mechanical properties of the different membranes, (**b**) the thermal stability of the QPSB-3 membrane before and after quaternization.

Another crucial factor in the evaluation of AEMs is stability, which encompasses mechanical, thermal, and chemical stability in strong alkaline environments. The mechanical properties of these membranes are shown in Figure 5a. It can be seen that all the exemplary membranes have an excellent tensile strength exceeding 40 MPa. As the content of stereo-contorted units increases, the tensile strength of the membrane is further enhanced while the elongation ratio at break decreases. This is because the stereo-contorted units raise the overall rigidity of the polymer backbone. Figure 5b illustrates the thermal stability of QPSB-3 membrane before and after quaternization. As shown, the temperature at which the polymer backbone starts to degrade severely is around 400 °C. After quaternization, the weight loss below 100 °C may be caused by the evaporation of residual moisture in the membrane. The weight loss corresponding to group degradation is found to occur above 200 °C. These results indicate that the membrane achieves sufficient mechanical and thermal stability.

**Figure 6:**
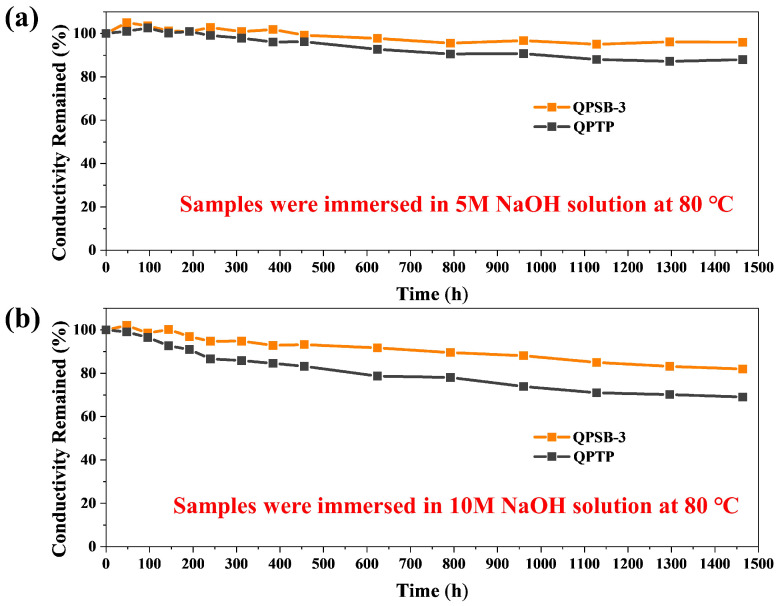
The conductivity change of QPSB-3 and QPTP membrane treated in (**a**) 5 M and (**b**) 10 M NaOH solution at 80 °C.

The chemical stability of membrane is evaluated by immersing samples in a highly concentrated alkaline solution. In order to investigate the influence of the ionic group position on the chemical stability, a quaternized poly(terphenyl piperidinium) (QPTP) membrane is used for comparison. This membrane differs from the QPSB-3 membrane in that the piperidinium is embedded into the backbone. As shown in Figure 6a,b, both two membranes display good chemical stability when immersed in a 5 M alkaline solution, with a conductivity loss of approximately 10% after 1500 h. However, when the concentration of alkaline in solution is further increased to 10 M, the QPTP membrane takes on a conductivity loss of approximately 30% after 1500 h of testing, whereas the QPSB-3 membrane has only about 18%. This is due to the fact that the piperidinium in the QPSB-3 membrane is located at the twisted sites of the polymer backbone. The twisted backbone structure helps to reduce the squeezing effect on piperidinium rings and therefore provides higher chemical stability. 

### 2.6. Fuel Cell Performance

**Figure 7:**
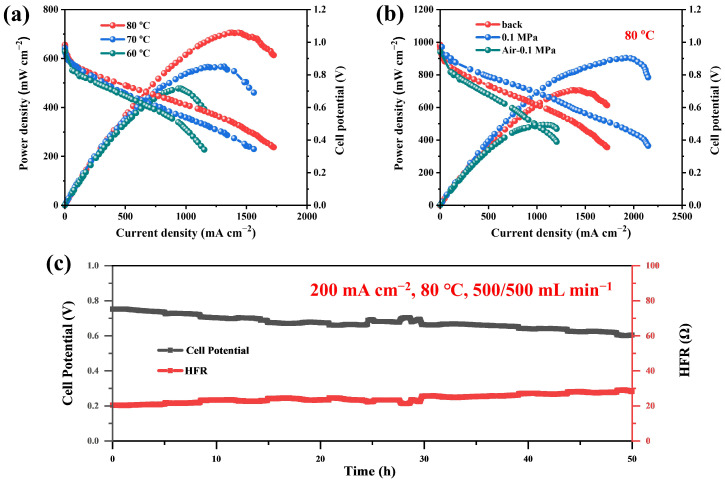
(**a**) The power density curves at different temperatures of the fuel cell assembled with the QPSB-3 membrane. (**b**) The power density curves at different background pressure and feeding gases. (**c**) The in situ stability testing of the fuel cell assembled with the QPSB-3 membrane.

To explore the actual application potential of the membrane, a fuel cell assembled using QPSB-3 membrane is tested for evaluation of its performance under different operating conditions. As shown in Figure 7a, raising the temperature can result in a notable increase in power density, namely from 477.3 mW cm^−2^ to 705.6 mW cm^−2^. This is attributed to improved mass transfer and facilitated catalytic activity. Figure 7b illustrates the impact of varying pressures and feeding gases on the fuel cell performance. It can be seen that an elevation of the operating pressure can further enhance the fuel cell performance. Ultimately, the fuel cell with a QPSB-3 membrane can reach an optimal power density of 904.2 mW/cm^2^ at 80 °C and 0.1 MPa. Moreover, the performance of the fuel cell under a H_2_/air feed condition is also evaluated. A power density of approximately 500 mW cm^−2^ is attained in this scenario.

In situ stability testing of the fuel cell is performed at a current density of 200 mA cm^−2^, a temperature of 80 °C and no background pressure. As shown in Figure 7c, the fuel cell operates continuously for more than 50 h, maintaining a cell potential above 0.6 V throughout. These results prove the good prospects for effective application in fuel cells of the membranes prepared in this work.

## 3. Conclusions

In this study, stereo-contorted monomers are incorporated to form a twisted all-carbon backbone via the superacid catalysis reaction. Piperidinium groups are dangled at the twisted sites of the backbone through the Suzuki coupling reaction, rather than being embedded into the main chains. The non-coplanar structure promotes ion conduction and reduces the squeezing effect of the polymer backbone on piperidinium rings. The Suzuki coupling reaction prevents the generation of any sensitive sites. Consequently, the AEMs as prepared exhibits good water uptake properties, OH^−^ conductivity and chemical stability. Fuel cells assembled using such advantageous membranes are also evaluated and prove good performance. Therefore, the designed membrane structure holds a promising future for broad applications in AEMFCs.

## Data Availability

Data is contained within the article and Supplementary Materials.

## Supplementary Materials

The following supporting information can be downloaded at: https://www.mdpi.com/article/10.3390/membranes14060121/s1, Figure S1: The ^1^H NMR spectrums of PSB and QPSB polymers; Figure S2: (a–c) the surface SEM image of QPSB-1, QPSB-2 and QPSB-3 membranes, (d–f) the cross-section SEM image of QPSB-1, QPSB-2 and QPSB-3 membranes; Figure S3: Conductivity comparison of the membranes prepared in this work with recently reported piperidine-based membranes with similar IEC [55,56,57,58,59,60,61]. Table S1: The property comparison of different membranes; Table S2: Property comparison of the membrane prepared in this work with recently reported piperidine-based membranes with similar IEC [55,56,57,58,59,60,61].
