# Supplementary material for: Anion Exchange Membrane with Pendulous Piperidinium on Twisted All-Carbon Backbone for Fuel Cell
Source: Membranes (Basel). 2024 May 23;14(6):121. doi: 10.3390/membranes14060121 (PMC11205426; doi:10.3390/membranes14060121)
Supplement: Supplementary file 1 [file membranes-14-00121-s001.zip › membranes-2981715-supplementary.pdf]

Supplementary Materials: Anion Exchange Membrane with Pendulous Piperidinium on Twisted All-Carbon Backbone for Fuel Cell

Huaqing Zhang, Wanjie Song, Lixuan Sun, Cui Yang, Xin Zhang, Mingyue Wu, Liang Wu, Xiaolin Ge * and Tongwen Xu *

Key Laboratory of Precision and Intelligent Chemistry, School of Chemistry and Materials Science, University of Science and Technology of China, Hefei 230026, China; zhanghq1@mail.ustc.edu.cn (H.Z.); swanjie@mail.ustc.edu.cn (W.S.); sunlixuan@mail.ustc.edu.cn (L.S.); yangcui1020@mail.ustc.edu.cn (C.Y.); aa123456@mail.ustc.edu.cn (X.Z.); mingyue_wu@mail.ustc.edu.cn (M.W.); liangwu8@ustc.edu.cn (L.W.)

* Correspondence: gexl@ustc.edu.cn (X.G.); twxu@ustc.edu.cn (T.X.)

Abstract: As a central component for anion exchange membrane fuel cells (AEMFCs), the anion exchange membrane is now facing the challenge of further improving its conductivity and alkali stability. Herein, a twisted all-carbon backbone is designed by introducing stereo-contorted units with piperidinium groups dangled at the twisted sites. The rigid and twisted backbone improves the conduction of hydroxide and brings down the squeezing effect of the backbone on piperidine rings. Accordingly, an anion exchange membrane prepared through this method exhibits adapted OH^- conductivity, low swelling ratio and excellent alkali stability, even in high alkali concentrations. Further, a fuel cell assembled with a such-prepared membrane can reach a power density of 904.2 mW/cm^2 and be capable of continuous operation for over 50 h. These results demonstrate that the designed membrane has good potential for applications in AEMFCs.

Keywords: anion exchange membrane; all-carbon backbone; twisted structure; free volume; fuel cell

1. Experimental

1.1. Materials

2,2'-Dibromo-9,9'-spirobi[fluorene], 1,1,1-Trifluoroacetone, dichloromethane, Trifluoromethanesulfonic acid (TFSA) and triflfluoroacetic acid (TFA) were obtained from Sinopharm Chemical Reagent Co., Ltd. 1-N-Boc-4-Methylene-piperidine, 9-Borabicyclo[3.3.1]nonane (9BBN, 0.5 M in THF), toluene, KOH, K_2CO_3, and Tetrakis(triphenylphosphine)palladium(0) ($Pd(PPh_3)_4$) were purchased form Sigma-Aldrich. N-methyl-2-pyrrolidone (NMP), Dimethyl sulfoxide (DMSO), acetone and iodomethane were obtained from China National Pharmaceutical Group Corporation. All chemicals were of analytical purity and utilized as received without any further purification.

1.2. Synthesis of Twisted Poly (Spirobifluorene-Biphenyl) (PSB) Polymer

The twisted backbone polymer was synthesis via a superacid catalyzed reaction. A total of 2.85 g of 2,2'-Dibromo-9,9'-spirobi[fluorene] and 0.82 g of 1,1,1-Trifluoroacetone were added to 6 ml of dichloromethane. Then, the mixture was cooled down to 0°C in an ice-water bath, and 9 ml of TFSA was added drip by drip. After 48 h of the reaction, another mixture was added which was formed of 0.82 g of 1,1,1-Trifluoroacetone and 0.93 g of biphenyl in 6 ml of dichloromethane. Then, another 9 ml of TFSA was subsequently added, and the reaction continued for about 8 h. During the polymerization, the reactants remained in the ice bath. Finally, the white precipitate was obtained by pouring the reaction solution into ethanol. The obtained polymer was washed repeatedly and dried in an oven. In this work, the proportions

of the 2,2'-Dibromo-9,9'-spirobi[fluorene] units are 30%, 40%, and 50%, respectively. The corresponding polymers are indicated by PSB-1, PSB-2, and PSB-3, respectively.

1.3. Functionalization of PSB Polymer through the Suzuki Coupling

A total of 1.6 g of 1-N-Boc-4-Methylene-piperidine was added in 16 mL of the solution of 9BBN 0.5 M in THF. Reaction mixture 1 was stirred for 2 h in the ice bath and then stirred at RT for an additional 2 h under the protection of N_2. In total, 1 g of PSB polymer was dissolved in 80 ml of toluene. A total of 0.8 g of KOH, 0.2 g of $Pd(PPh_3)_4$ and 15 mL deionized (DI) water were added into the solution to form reaction mixture 2. The round flask was repeatedly degassed and equipped with condensate and a N_2 inlet before reaction mixture 1 was added into reaction mixture 2. The reactions were performed for 48 h at 80 °C. After cooling to room temperature, the mixture was precipitated in methanol, washed with methanol and water, and completely dried in oven at 60 °C. The obtained polymer was dissolved in a mixed solvent of NMP/DMSO/TFA and heated at 80 °C for 24 h to remove the BOC group. Finally, the functionalized PSB polymer was precipitated in acetone and dried at room temperature.

1.4. Quaternization and Preparation of Membrane

The synthesized polymer was dissolved in NMP/DMSO solution (1:1) at 80 °C. Then, the mixed solution was cooled down to 40 °C and ten times the equivalent of iodomethane was added. The K_2CO_3 was used as the acid binding agent and the reaction was carried out under light protection for 7 d. The product was precipitated in acetone and washed thoroughly with acetone and water. The quaterinized polymer was dried and dissolved in the NMP/DMSO solution. The membrane was prepared by casting the solution onto a square glass plate and heated at 80 °C. After the solvent has completely evaporated, the membrane was peeled off the glass plate. These membranes were named QPSB-x. Before use, these membranes were immersed in 1 M NaOH solution for 24 h to convert them to the OH^- form.

1.5. Characterizations

The ^1H NMR spectra of all prepared products were recorded at nuclear magnetic resonance spectroscopy (^1H NMR, Bruker 510 instrument, 400 MHz). The surface, cross-section morphology images of the membranes were obtained by scanning electron microscopy (SEM, Nova NanoSEM 450). The carbon dioxide (CO_2) sorption and desorption experiments were performed with a specific surface and aperture analyzer (ASAP 2020, Micromeritics, USA). The glass-transition temperature (Tg) was measured using a Mettler DSC 822 under N2. An atomic force microscope (AFM) was used to reveal the microphase separation of membranes. Small-angle X-ray scattering (SAXS) analysis was recorded on an Anton Paar Saxesess mc2 instrument equipped with an Osmic microfocus Cu K_α source and a parallel beam optic. Mechanical properties measurements of the as-prepared AEMs were carried out using a discovery DMA 850 dynamic mechanical analyzer (TA Instruments Co., USA). The thermal stability of AEMs was measured using a TGA Q5000 instrument in the temperature range of 30–800 °C at a heating rate of 10 °C min^{-1}.

1.6. Property Measurements

The IEC of membrane samples was measured using the Mohr titration method. The conductivity of membranes samples at different temperatures were measured using a standard four-point probe technique on an Auto lab Zahner Zennium E (Germany) in the galvanostatic mode.

The water uptake (WU) and swelling ratio (SR) were determined by the difference of the weights and lengths of membranes in dry and wet states, respectively. Firstly, the weights and lengths of the dry samples were recorded (W_{dry}, L_{dry}). Then, the samples were immersed in 1M

NaOH for 2 h at different temperatures. Residual NaOH aq. was washed by DI water, and the wet weights and lengths (W_{wet}, L_{wet}) of the samples were measured. The WU and SR were calculated using the following equation:

$$WU = \frac{W_{wet} - W_{dry}}{W_{dry}} \times 100\%$$

$$SR = \frac{L_{wet} - L_{dry}}{L_{dry}} \times 100\%$$

The chemical stability of membranes was measured by immersing the membranes in 5 M or 10 M NaOH solutions at 80 °C for different times. The changes in conductivity were monitored.

The λ is calculated from the water absorption and IEC of the membrane, which represents the number of water molecules per mol of charged functional groups. The formula is as follows:

$$\lambda = \frac{100 \times WU}{18 \times IEC}$$

1.7. H2/O2 Single Cell Performance

The Pt/C (Johnson Matthey HiSpec 4000, 40 wt% Pt) and Pt/Ru/C (Johnson Matthey HiSpec 10,000, 40 wt% Pt and 20 wt% Ru) were used as the cathode and anode electrode catalysts, respectively. The Quaternized Poly(arylene piperidinium) polymers were dissolved in DMSO to prepare a 5% ionomer solution. Subsequently, the ionomer solution and catalysts were added to an isopropanol (IPA)/deionized (DI) water (9 to 1) solution to prepare the catalyst inks. The mass ratios of the ionomer:carbon:catalyst at the anode and cathode were fixed at 1: 1.33: 2 and 1: 2: 1.33, respectively. The thickness of the membranes used in fuel cell testing is 25 ± 5 μm. After homogeneous dispersion by ultrasonication, the ink was sprayed onto the AEM to prepare the membrane electrode assembly (MEA). The metal loading in both the anode and cathode was controlled at 0.5 mg cm^{-2}, and the electrode area was 5 cm^2. Before testing, the prepared MEA was immersed in 1M NaOH solution for 24 h to convert it to OH^- form. An 850E Multi Range fuel cell test station (Scribner Associates, USA) was used to test the AEM. The fuel cell was tested at different temperature and pressure conditions. H_2/O_2 or H_2/air were used as the feed gas with a feed rate of 500 mL min^{-1}. After the cell stabilized, the cell voltage and power density at each current density were recorded. The in situ stability tests were carried out at 200 mA cm^{-2} current density and 80 °C.

2. Supplementary Data

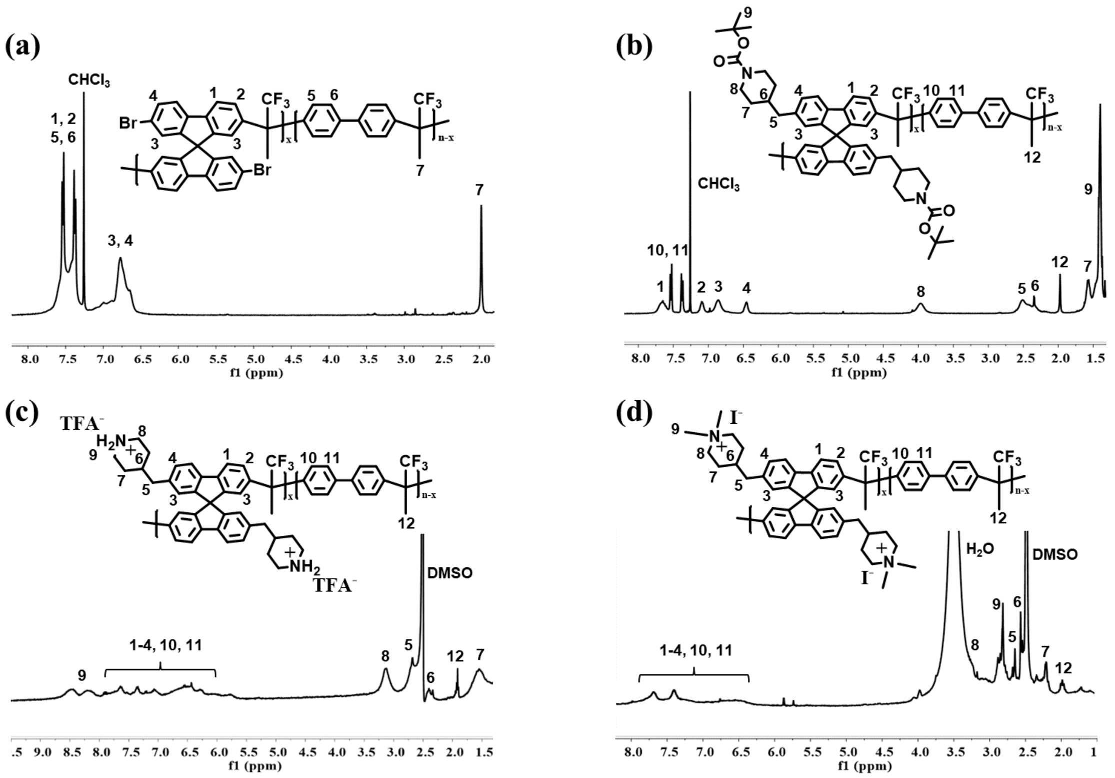

Figure S1. The ^{1}H NMR spectrums of PSB and QPSB polymers

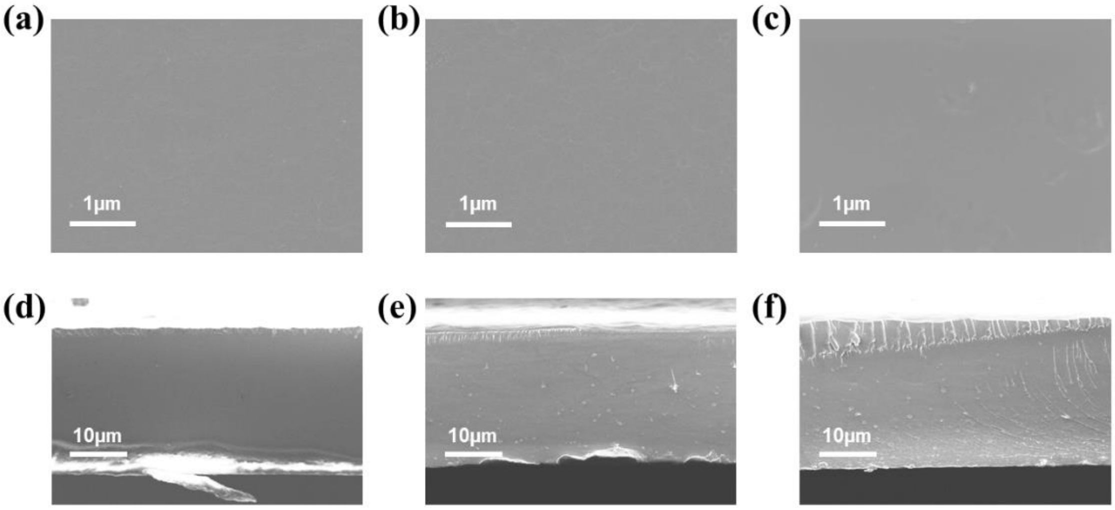

Figure S2. (a–c) The surface SEM image of QPSB-1, QPSB-2 and QPSB-3 membranes; (d–f) the cross-section SEM image of QPSB-1, QPSB-2 and QPSB-3 membranes.

Table S1. The property comparison of different membranes.

Membrane	IEC (mmol g^{-1})	WU (wt. %)	SR (%)	δ (Cl^{-}) (mS cm^{-1})		Tensile Strength (MPa)
				30 °C	80 °C	
**QPSB-1**	1.38	27.34	5.9	20.89	49.09	44.01
**QPSB-2**	1.65	44.01	6.5	40.24	73.63	50.45
**QPSB-3**	1.81	55.56	7.3	55.21	99.44	53.29

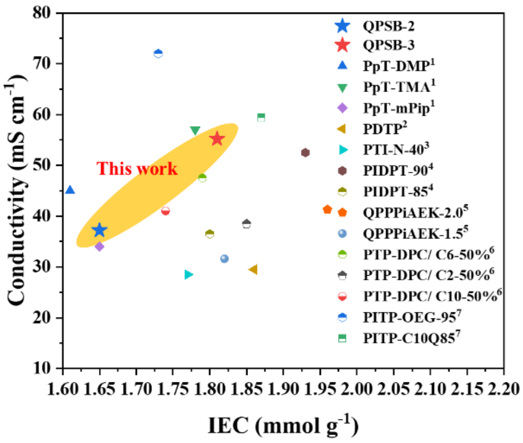

Figure S3. Conductivity comparison of the membranes prepared in this work with recently reported piperidine-based membranes with similar IEC [55–61].

Table S2. Property comparison of the membrane prepared in this work with recently reported piperidine-based membranes with similar IEC [55–61].

Membranes	IEC (mmol g^{-1})	Conductivity (mS cm^{-1})	WU (%)	SR (%)	Mechanical strength (MPa)	Chemical stability		Power density (mW cm^{-2})	Reference
						alkaline environment	Test duration		
QPSB-3	1.81	55.2	55.6	7.3	53.3	10M NaOH, 80°C	1500 h	904	This work
QPSB-2	1.65	37.2	44.0	6.5	50.4	—	—	—	This work
PpT-DMP	1.61	45.0	38.0	18.0	—	10M NaOH, 90°C	240 h	—	[1]
PpT-TMA	1.78	57.0	35.0	9.5	—	5M NaOH, 90°C	240 h	—	[1]
PpT-mPip	1.65	34.0	31.0	6.5	—	5M NaOH, 90°C	240 h	—	[1]
PDTP	1.86	29.5	72.1	17.6	28.5	2M NaOH, 80°C	720 h	214	[2]
PTI-N-40	1.77	28.5	11.5	7.7	30.9	2M KOH, 80°C	800 h	180	[3]
PIDPT-90	1.93	52.5	15.2	6.3	44.0	2M NaOH, 80°C	1632 h	75.6	[4]
PIDPT-85	1.80	36.5	14.5	5.7	40.6	2M NaOH, 80°C	1632 h	29.8	[4]
QPPPiAEK-2.0	1.96	41.3	94.9	27.1	35.1	1M KOH, 80°C	700 h	187.7	[5]
QPPPiAEK-1.5	1.82	31.6	65.1	29.2	41.5	1M KOH, 80°C	1000 h	174.2	[5]
PTP-DPC/C6-50%	1.79	47.5	20.5	7.8	36.5	2M NaOH, 80°C	1080 h	616	[6]
PTP-DPC/C2-50%	1.85	38.5	11.2	4.9	31.5	2M NaOH, 80°C	1080 h	—	[6]
PTP-DPC/C10-50%	1.74	41.0	30.9	8.9	40.2	2M NaOH, 80°C	1080 h	—	[6]
PITP-OEG-95	1.73	72.0	22.8	14.3	51.5	1M NaOH, 80°C	1080 h	1230	[7]
PITP-C10Q85	1.87	59.4	43.0	17.9	55.1	1M NaOH, 80°C	1080 h	621	[7]

References

55. Chen, S.; Pan, D.; Gong, H.; Jannasch, P. Hydroxide Conducting Membranes with Quaternary Ammonium Cations Tethered to Poly(arylene alkylene)s via Flexible Phenylpropyl Spacers. *Chem. Mater.* **2023**, *36*, 371-381.
56. Huang, J.; Yu, Z.; Tang, J.; Wang, P.; Zhang, X.; Wang, J.; Lei, X. A non-cationic crosslinking strategy to improve the performance of anion exchange membranes based on poly(aryl piperidinium) for fuel cells. *Colloid. Surface. A* **2023**, *674*, 131890.
57. Gu, Y.; Zhang, Y.; Wang, Z.; Liu, D.; Wang, Y.; Dong, T.; Wang, S.; Li, Z.; Wu, J.; Lei, Y. Synergistic functionalization of poly(p-terphenyl isatin) anion exchange membrane with quaternary ammonium and piperidine cations for fuel cells. *Ind. Chem. Mater.* **2024**, *2*, 141-153.
58. Wang, Y.; Wang, Y.; Guo, M.; Ban, T.; Zhu, X. Poly(isatin-piperidinium-terphenyl) anion exchange membranes with improved performance for direct borohydride fuel cells. *Int. J. Hydrog. Energy* **2023**, *48*, 14837-14852.
59. Qi, L.; Wang, X.; Chao, G.; Li, N.; Zhang, X. Enhancement in the alkaline stability of poly(arylene ether ketone)-typed anion-exchange membranes via phenylene piperidinium blocks. *J. Membr. Sci.* **2023**, *687*, 122057.
60. Liu, Y.J.; Gao, W.T.; Zhu, A.M.; Zhang, Q.G.; Liu, Q.L. High-performance di-piperidinium-crosslinked poly(p-terphenyl piperidinium) anion exchange membranes. *J. Membr. Sci.* **2023**, *687*, 122045.
61. Zhang, J.; Ma, W.; Yin, T.; Chen, S.; Zhang, X.; Li, N.; Liu, L. Oligo (ethylene glycol)-grafted poly(terphenyl indole piperidinium) with high water diffusivity for anion exchange membrane fuel cells. *J. Membr. Sci.* **2024**, *694*, 122424.
